# Megaendoprostheses in the management of malignant tumors of the lower extremities—risk factors for revision surgery

**DOI:** 10.1186/s13018-021-02654-5

**Published:** 2021-08-18

**Authors:** Moritz von Salis-Soglio, Mohamed Ghanem, Christian Lycke, Andreas Roth, Georg Osterhoff

**Affiliations:** Department of Orthopaedics, Trauma and Plastic Surgery, University Hospital Leipzig, Liebigstr. 20, 04103 Leipzig, Germany

**Keywords:** Tumor, Cancer, Megaimplants, Total hip replacement, Total knee replacement, Endoprosthesis, Complications, Risk factors

## Abstract

**Abstract:**

**Background:**

Improved oncological and surgical measures now enable curative treatment of malignant lower extremity tumors in majority of cases. Complication rates associated with surgical resection of lower extremity tumors and replacement with megaendoprostheses are high. The aim of this study was to identify risk factors that predispose to revision surgery following the use of megaimplants in curative treatment of malignant tumors of the lower extremities.

**Methods:**

this retrospective study included patients aged ≥ 18 years who underwent implantation of a megaendoprosthesis for tumors or metastatic lesions of the lower extremities between January 2010 and December 2020. Baseline characteristics and information on adjuvant treatment, hospitalization time, comorbidities, mobility, complications, and revision surgery were considered. Primary outcomes were revision surgery and reasons for revision. Secondary outcomes were in-hospital complications and the duration of hospitalization.

**Results:**

Fifty-four patients (48% female, age 63 years, SD 15) were available for final analysis. Surgeries were performed at hip level in 37 patients (68.5%) and at knee level in 17 patients (31.5%). Revision for wound-related causes was performed in 12 cases (22.2%), with microbiological proof of infection in 8 cases (14.8%). Revision for hip joint instability was carried out in 4 cases (7.4%) and for disconnection between components of the megaimplant in 2 cases (3.7%). Those patients requiring a wound-related revision had undergone a longer primary surgical intervention than those who required an implant-related revision (276 vs 134 min, *p* = .002). Wound drains after the primary implantation remained longer in situ in patients who later required revision surgery for wound-related complications (5 vs 3 days, *p* = .020). An ASA > 3 was associated with an increased likelihood for in-hospital complications in general (*p* = .041), and in-hospital death in particular (*p* = .012).

**Conclusions:**

The management of malignant tumors of the lower extremities with megaendoprostheses is associated with a high rate of wound-related complications. Swift surgical performance and early postoperative removal of wound drains minimize the risk of complications in general and the necessity of revision surgery in particular. Patients with more comorbidities were more likely to suffer in-hospital complications.

## Introduction

The demographic shift toward an elderly population is inevitably linked to an increase in the incidence of malignant primary tumors and metastases in the musculoskeletal system [8, 14]. A few decades ago, the treatment of malignant bone tumors involved amputation of the affected limb. The introduction of megaimplants as an alternative to amputation therefore represented a milestone of particular relevance to the treatment of malignant bone tumors of the lower extremities [14, 26].

Metastases manifest with different rates of incidence in the long bones, most commonly in femur, humerus, and tibia [5], particularly in metadiaphyseal sections.

The improved (neo-) adjuvant chemotherapy, radiotherapy and surgical measures using modular megaimplants have made an in sano resection of tumors with concommitant extremity preservation possible in the vast majority (approx. 90%) of cases [1, 6, 14, 24, 26].

The overall survival rate after resection of primary malignant bone tumors has been improved in recent years and now ranges from 61 to 92%. Similarly, the survival time of those patients who develop skeletal metastases has also improved with 5-year survival rates currently approaching 30%) [1, 24].

Implantation of mega-endoprostheses in patients after tumor resection mainly serves to reduce pain, reconstruct the length of the extremity and restore mobility [11, 20].

Surgical management of malignant tumors of the lower extremities with skeletal reconstruction using mega-implants is a complex intervention that can expose multimorbid patients in particular to perioperative risks and complications [6, 8, 15, 26].

The relatively limited number of tumor endoprosthetic procedures, the variety of individual indications and the different modalities of additional oncological therapy such as (neo-)adjuvant chemotherapy and radiotherapy has led unavoidably to a significant degree of heterogeneity in cases included in scientific studies. This imposes an important limitation the number of studies and classifications available [13] and has resulted in the vast majority of studies of megaendoprostheses being focused on analyses of post-surgical complications such as infection, dislocation, aseptic loosening and disconnection of modular parts [6, 8, 10, 14, 26].

This study was designed to identify risk factors predisposing to revision surgery and to assess the overall rate of revision surgery after implantation of megaimplants in the context of curative surgery of malignant tumors of the lower extremities. Our aim is the informed adjustment of treatment strategies to minimize the need for revision surgery in the interests of both patient welfare and cost-effectiveness. These aspects are in line with context of translational orthopedics filing the gap between research and clinical practice [21].

## Material and methods

A monocentric retrospective cohort study was conducted at an academic orthopedic center. The protocol of this study was approved by the institutional ethics committee.

### Patients

Consecutive patients aged ≥ 18 years who underwent implantation of a megaendoprosthesis for tumors or metastatic lesions of a lower extremity between January 2010 and December 2020 with a minimum follow-up of 3 months were identified from the authors’ hospital information system. A retrospective chart review was performed for all patients identified as eligible. Patients with diaphyseal or total femur replacement were excluded, as were those who had expressed objection to the use of their data for research purposes.

Baseline characteristics and information on adjuvant treatment, hospitalization time, comorbidities, mobility, complications, and revision surgery were obtained. The ASA physical status classification system (ASA) of the American Society of Anesthesiologists was documented for all patients.

### Outcome

Primary outcomes were revision surgery and reasons for revision. Secondary outcomes were in-hospital complications and duration of the hospitalization.

### Statistical analysis

Statistical analysis was performed in SPSS 25.0 (SPSS Inc., Chicago, IL, USA). Unless otherwise denoted, data were summarized as mean and standard deviation (SD).

Nominal variables were associated using chi-square or Fisher’s exact tests and non-parametric tests were used to compare continuous data. To determine the prognostic value of potential factors identified in the exploratory analysis, a binary logistic regression analysis was performed and odds ratios with a confidence interval (CI) of 95 % were calculated. The level of statistical significance was set at *p* < 0.05.

## Results

Fifty-four patients with tumor-endoprostheses (26 females, 48%) were available for final analysis. The mean age was 63 years (SD 15, range, 19 to 81) and mean follow-up was 18 months (SD 25, range, 3 to 113). Thirty-one patients (57.4 %) had a malignant primary bone tumor, while a metastatic bone lesion was resected in 23 cases (42.6%). Metastatic disease was present in 34 patients (63.0 %).

Most patients had pre-existing comorbidities with cardiovascular diseases being the most frequent (74.0%), followed by diseases of the lung (20.4%), and diabetes mellitus (18.5%). The median ASA was 3 (range, 1 to 4) with 28 patients scaled ASA 3 (51.9%), 22 scaled ASA 2 (40.7%), 3 scaled ASA 4 (5.6 %), and 1 patient with an ASA of 1 (1.9%).

**Fig. 1:**
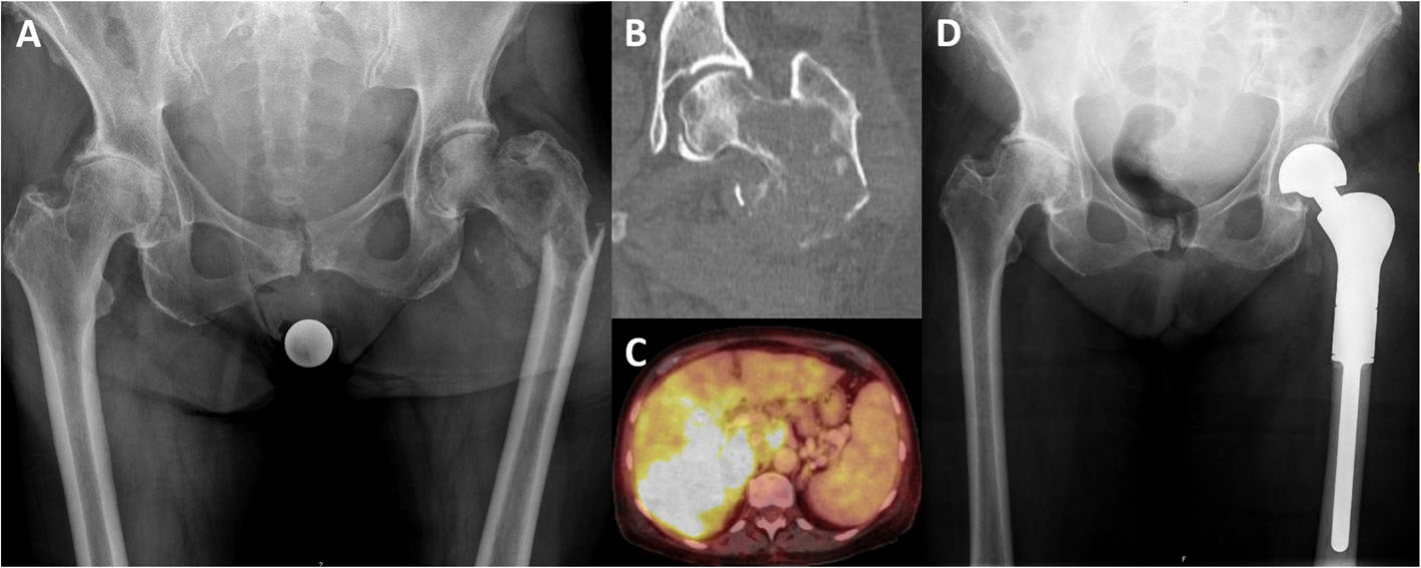
Case of a 70-year-old female patient with a pathological proximal femur fracture due to a metastatic lesion of a cholangiocellular carcinoma (CCC, UICC Stadium IV). **A** Conventional radiography showing pathological fracture of the left femur. **B** Computed tomography (CT) shows a lytic lesion of the proximal femur. **C** PET-CT scan with a large malignant tumor of the liver that histologically revealed to be a CCC. **D** Postoperative antero-posterior radiograph after resection and replacement of the proximal femur

**Fig. 2:**
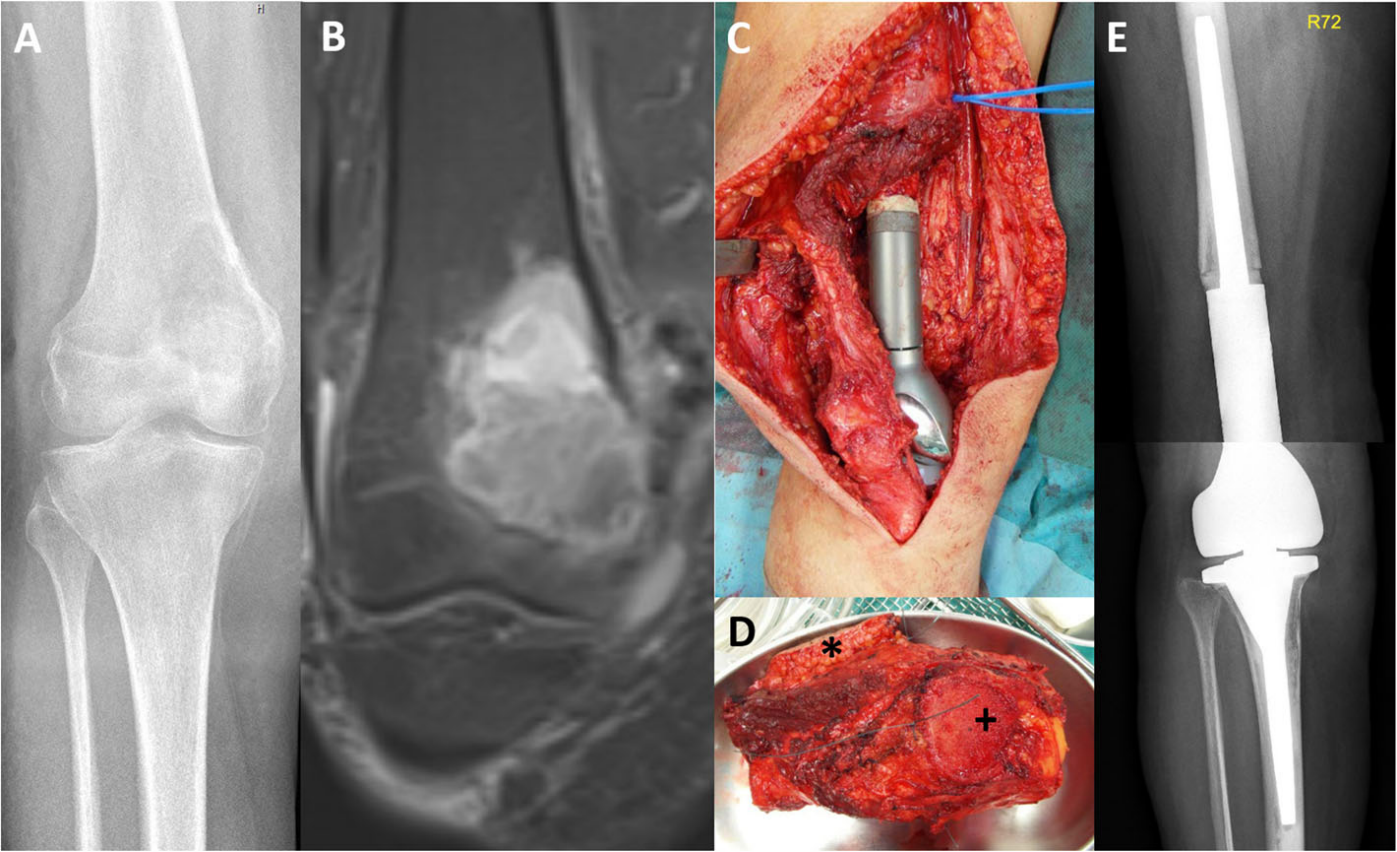
Case of a 75-year-old female patient with a Ewing-like sarcoma of the right distal femur with infiltration of the joint space. **A** Conventional radiography showing the osteolytic lesion. **B** Magnetic resonance imaging with contrast agent shows erosion of the articular cortex of the medial femur condyle. **C** Intraoperative photograph after extra-articular knee resection and implantation of a megaimplant. The patella was split in the coronal plane. **D** Resected knee (top: medial, left: proximal) with the articular part of the patella (*) and the biopsy scar (+) attached to it. **E** Postoperative antero-posterior radiograph

Surgeries were performed at hip level in 37 patients (68.5%) and at knee level in 17 patients (31.5%, Figs. 1 and 2). The mean duration of the tumor resection and primary implantation of a megaendoprosthesis was 229 min (SD 111) and the mean length of hospitalization was 19days (SD 13, range, 6 to 72). Wound drains were applied intraoperatively in 50 patients (92.6%) and removed after mean 4 days (SD 2, range, 1 to 7).

### Revision surgery

**Fig. 3:**
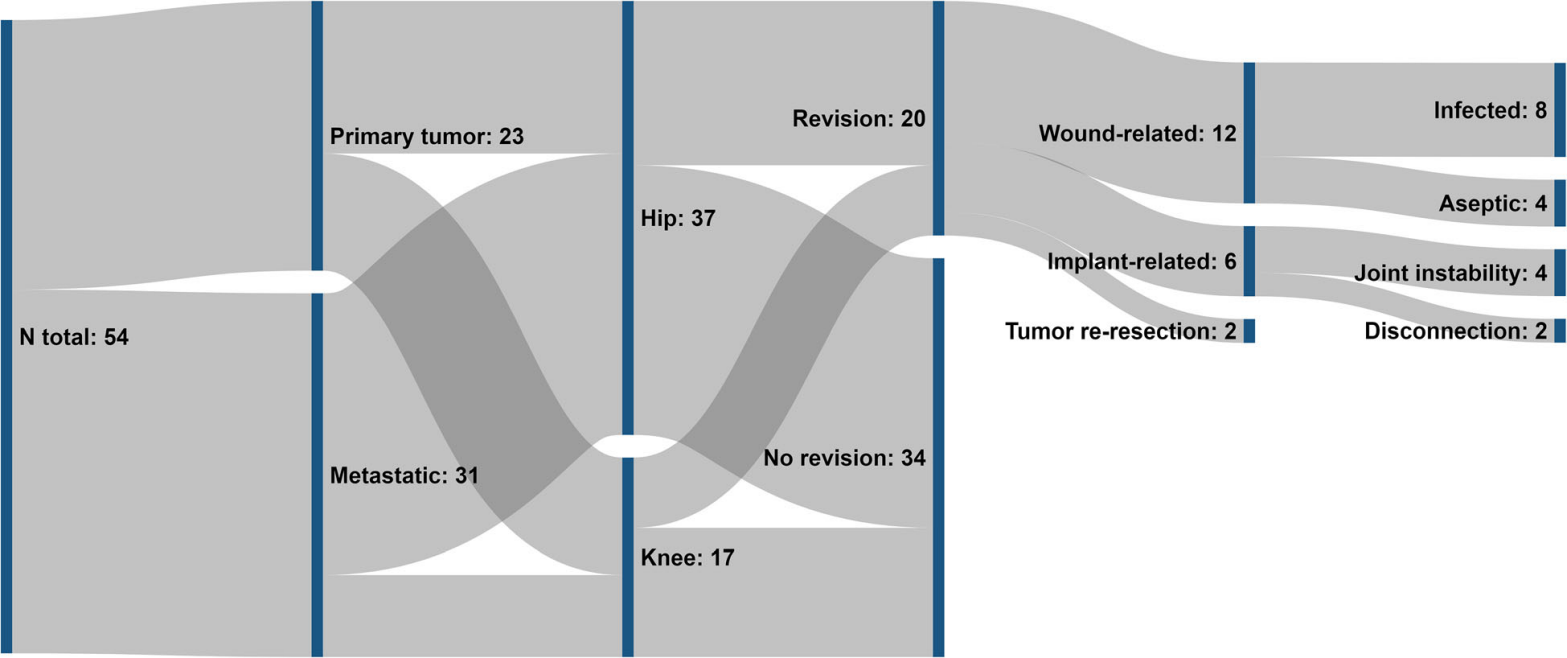
Flow chart of complications

Revision surgery was necessary in 18 patients (33.3%) after median 29 days (range, 9 to 3195 days, Table 1, Fig. 3).

**Table 1:** Reasons for and type of revision surgeries

	Age	Sex	Type of implant	Reason for revision	Type of revision
**Implant-related**	71	Male	Distal femur	Functional due to mechanical problem with connector	Change of connector and inlay
	77	Female	Prox. femur	Hip dislocation	Open reduction, change of head
	53	Female	Prox. femur w/ THA	Hip dislocation	Closed reduction
	44	Female	Prox. femur w/ THA	Hip dislocation	Change of acetabular component, constrained inlay
	72	Male	Prox. femur	Hip dislocation	Open reduction, change of head
	51	Female	Dist. femur	Arthrofibrosis	Arthrolysis, change of connector and inlay
**Wound-related**	71	Male	Prox. femur	SSI	Lavage, change of modules, stem left in place
	72	Male	Prox. femur	SSI	Lavage, change of inlay
	77	Female	Dist. femur	SSI	Lavage, change of inlay
	78	Male	Prox. femur	SSI	Lavage, change of inlay
	70	Female	Dist. femur	SSI	Lavage, implant removal, spacer
	78	Male	Prox. femur	SSI	Lavage, implant removal, spacer
	77	Male	Dist. femur	SSI	Lavage, change of inlay
	71	Female	Prox. femur	SSI	Lavage, change of inlay
	53	Female	Prox. femur	Wound seroma	Lavage
	44	Male	Dist. femur	Wound seroma	Lavage, change of inlay
	67	Male	Prox. femur	Hematoma	Lavage
	37	Male	Acetabular replacement	Wound dehiscence	Free flap coverage
**Tumor-related**	71	male	Acetabular replacement	Local recurrence	Hip exarticulation
	75	female	Dist. femur	R1 resection	Re-resection

Revision for wound-related causes was performed in 12 cases (22.2%), with microbiological proof of infection in 8 cases (14.8%). Revision for implant-related causes was carried out in 6 cases (11.1%), with hip joint instability in 4 cases (7.4%) and disconnection within the megaimplant in 2 cases (3.7%). No revisions for implant loosening were necessary during the observed follow-up interval. In 2 patients (3.7%) histological examination revealed an insufficient safety margin after tumor resection. Therefore, revision surgery was performed with subsequent histological proof of in sano resection.

Patients requiring a wound-related revision had undergone a primary surgical intervention that was significantly longer than that for patients requiring an implant-related revision (276 vs 134 min, *p* = .002).

Wound drains after the primary implantation remained in situ longer in those patients who later required revision surgery for wound-related complications (5 days, SD 2) than they did either in patients who required implant-related revisions (3 days, SD 1, *p* = .020) or in all patients without a wound-related complication (4 days, SD 1, *p* = 0.014). Logistic regression analysis revealed that each additional day of leaving the drains in situ increases the likelihood of a revision for wound-related complications by 112% (coefficient *B* = 0.752, OR 2.121, 95% CI 1.128, 3.987)

### In-hospital complications

In-hospital complications not directly related to the surgical wound or the implant itself were seen in 7 patients (13.0%). This included 2 cases with cardiovascular events (3.7%), 2 pulmonary embolisms (3.7 %), 2 cases with pneumonia (3.7%), and 1 urinary tract infection (1.9%). These complications were lethal in 4 (7.4%) patients. No other in-hospital complications were observed.

Patients who died during the hospitalization were significantly older (mean 76 years, SD 6 vs. 61 years, SD 15, *p* = .039). Patients with pre-existing pulmonary diseases were more likely to have an in-hospital pneumonia (*p* = .038), and patients under ongoing chemotherapy during the surgery were more likely to have a cardiovascular event (*p* = .031). An ASA > 3 was associated with an increased likelihood for in-hospital complications in general (*p* = .041), and in-hospital death in particular (*p* = .012). Logistic regression analysis revealed that patients with an ASA > 3 had an odds ratio of 0.020 (95% CI 0.001, 0.331, coefficient *B* = − 3.892) for death during hospitalization.

### Hospitalization time

The duration of the initial surgery showed a strong correlation with the duration of the entire hospital stay (Pearson’s *r =* .614, *p* < .001), while there was only a very weak association between the time to wound drain removal and hospital stay (Pearson’s *r =* .321, *p* = .043).

## Discussion

In this study, the observed overall rate of revision surgery after implantation of megaimplants in the management of tumors of the lower extremity was 33.3% after median 29 days (range, 9 to 3195 days).

Most studies published to date have focused on the rate of a particular complication such as dislocation, infection, loosening or disconnection of modular parts [2, 4, 6, 8, 9, 12, 17, 18, 22, 26–28]. To the best of our knowledge, only few studies have evaluated the overall rate of revision surgery after resection of malignant tumors of the lower extremity with subsequent reconstruction with megaendoprostheses [25].

Implant-specific complications, such as disconnection or material breakage occurred rarely in the patient population analyzed here (3.7%), in line with similar studies reported in literature [26]. Despite being an initial concern, the disconnection of modular parts no longer plays an essential role in large megaendoprosthesis systems [25, 26].

In 4 of our cases (7.4%), we performed revision surgery due to joint instability of the hip after proximal femoral replacement. Rates of dislocation between 2% and 28% have been reported in the literature, with the wide range reflecting heterogeneity both within and between the patient populations [3, 11, 17–19, 23, 29]. The dislocation rate of 7.4% in our cohort which is based on partial pelvic replacement, proximal femoral replacement, and total femoral replacement is comparable to that observed in other studies. Nonetheless, the course of treatment necessitated by dislocation can involve reoperations or orthotic fittings and is often a significant burden.

The greatest challenge in the case of megaendoprostheses, however, is undoubtedly periprosthetic infection, which usually entails an extremely time-consuming and costly treatment. In our study, revision for wound-related causes was performed in 12 cases (22.2%), with microbiological proof of infection in 8 cases (14.8%). Again, this is consistent with the infection rates between 3% and 36% that have been reported in the literature [2, 4, 6, 8, 9, 12, 17, 18, 22, 28]. Importantly, our analysis clearly identified a longer duration of surgery and the delayed removal of wound drains following primary surgery as risk factors for wound-related revision surgery after implantation of megaendoprostheses.

As may be expected, patients who had pre-existing comorbidities were more likely to suffer from in-hospital complications not directly related to the wound or the implant itself.

The limitations of this study lie in its retrospective nature, the inhomogeneity of the patient population, and the fact that we were not able to evaluate any pre- and postoperative scores for the patients. However, these same limitations apply to the vast majority of studies that dealt with megaimplants in general, and megaimplants in management of tumors of the lower extremity in particular.

Although the minimum follow-up period of 3 months must be considered as a further limitation of our study, the average follow-up period was 18 months. In view of this, the fact that no revision surgery was carried out due to loosening of implants is undoubtedly a positive aspect.

Analysis of the survival rate of megaendoprostheses is complicated by the heterogeneity of the patient population and the underlying clinical characteristics. However, an increasing rate of loosening is observed from proximal femur replacement to distal femur replacement to proximal tibia or total femur replacement that is consistent with degrees of biomechanical load [7, 16].

## Conclusion

Megaendoprostheses will undoubtedly continue to be of major relevance in tumor surgery of the lower extremity. They present technically feasible solutions in the most difficult situations involving large bone defects and usually facilitate the early mobilization and weight-bearing that is particularly important for the management of multimorbid patients.

Swift surgical performance and early postoperative removal of wound drains minimize the risk of complications in general and the necessity of revision surgery in particular.

Further studies with long-term follow-up are needed to identify further risk factors for complications and for revision surgery following tumor surgery of the lower extremity and reconstruction with megaimplants.

## Data Availability

Anonymized grouped data are available upon request from the corresponding author.

## Abbreviations

ASA: American Society of Anesthesiologists Scale
CCC: Cholangiocellular carcinoma
SD: Standard deviation
UICC: Union for International Cancer Control
